# Primary and specialist care interaction and referral patterns for individuals with chronic kidney disease: a narrative review

**DOI:** 10.1186/s12882-024-03585-z

**Published:** 2024-04-30

**Authors:** Clyson Mutatiri, Angela Ratsch, Matthew McGrail, Sree Krishna Venuthurupalli, Srinivas Kondalsamy Chennakesavan

**Affiliations:** 1 Renal Medicine, Wide Bay Hospital and Health Service, Bundaberg, QLD Australia; 2 Rural Clinical School, Faculty of Medicine, The University of Queensland, Bundaberg, QLD Australia; 3 Research Services, Wide Bay Hospital and Health Service, Hervey Bay, QLD Australia; 4 Rural Clinical School, Faculty of Medicine, The University of Queensland, Rockhampton, QLD Australia; 5 Kidney Service, Department of Medicine, West Moreton Hospital and Health Service, Ipswich, QLD Australia; 6 Faculty of Medicine, The University of Queensland, Brisbane, Australia; 7 Rural Clinical School, Faculty of Medicine, The University of Queensland, Toowoomba, QLD Australia; 8 Rural Clinical School, Faculty of Medicine, The University of Queensland, Hervey Bay, QLD Australia

**Keywords:** Chronic kidney disease, Timing of referral, Adequacy of pre-dialysis care

## Abstract

**Background:**

Timely referral of individuals with chronic kidney disease from primary care to secondary care is evidenced to improve patient outcomes, especially for those whose disease progresses to kidney failure requiring kidney replacement therapy. A shortage of specialist nephrology services plus no consistent criteria for referral and reporting leads to referral pattern variability in the management of individuals with chronic kidney disease.

**Objective:**

The objective of this review was to explore the referral patterns of individuals with chronic kidney disease from primary care to specialist nephrology services. It focused on the primary-specialist care interface, optimal timing of referral to nephrology services, adequacy of preparation for kidney replacement therapy, and the role of clinical criteria vs. risk-based prediction tools in guiding the referral process.

**Methods:**

A narrative review was utilised to summarise the literature, with the intent of providing a broad-based understanding of the referral patterns for patients with chronic kidney disease in order to guide clinical practice decisions. The review identified original English language qualitative, quantitative, or mixed methods publications as well as systematic reviews and meta-analyses available in PubMed and Google Scholar from their inception to 24 March 2023.

**Results:**

Thirteen papers met the criteria for detailed review. We grouped the findings into three main themes: (1) Outcomes of the timing of referral to nephrology services, (2) Adequacy of preparation for kidney replacement therapy, and (3) Comparison of clinical criteria vs. risk-based prediction tools. The review demonstrated that regardless of the time frame used to define early vs. late referral in relation to the start of kidney replacement therapy, better outcomes are evidenced in patients referred early.

**Conclusions:**

This review informs the patterns and timing of referral for pre-dialysis specialist care to mitigate adverse outcomes for individuals with chronic kidney disease requiring dialysis. Enhancing current risk prediction equations will enable primary care clinicians to accurately predict the risk of clinically important outcomes and provide much-needed guidance on the timing of referral between primary care and specialist nephrology services.

## Introduction

Chronic kidney disease (CKD) is a major non-communicable chronic disease whose burden continues to rise globally [1]. It is estimated that between 8% and 16% of the world’s population is living with indicators of CKD, such as increased urinary albumin excretion and a reduced estimated glomerular filtration rate (eGFR) [2]. In addition to increasing the risk for all-cause and cardiovascular mortality, CKD constitutes a major cost burden both in terms of direct healthcare systems expenditure and productivity losses from those living with CKD [3–6]. An analysis based on the Global Burden of Disease (GBD) study 2017 across 195 countries found that CKD resulted in 1.2 million deaths and was the 12th leading cause of death worldwide [7]. In Australia, the 1999–2000 Australian Diabetes, Obesity and Lifestyle Study (AusDiab) and the 2011–12 National Health Measurement Survey (NHMS), estimated that the total number of Australian adults ≥ 25 years of age with CKD increased by almost 50%, from 1 million in 1999–2000 to over 1.5 million people in 2011–2012 [8]. CKD was responsible for 11% of all Australian deaths in 2018, either as an underlying cause or as an associated cause [9].

Globally, several studies [10–13] suggest that timely referral of individuals with CKD to nephrology services portends favourable outcomes and hence ameliorates its impact. However, it is not possible for every patient with CKD to be managed through secondary care due to the limited nephrologist workforce and constrained fiscal resources. Worldwide shortages of the nephrology workforce have been highlighted extensively, with an overall density of nephrologists reported at 8.83 per million population [14–20]. However, there is considerable variation amongst different countries, with high-income countries demonstrating the highest nephrologist density while low-income countries showing the lowest density [19]. In Australia there are currently approximately 1.7 million individuals aged > 18 years with clinical evidence of CKD; specialist referral of all patients with CKD would see an average of > 2,800 patients per nephrologist [9, 21, 22]. In order to balance clinical need for secondary care against nephrology workforce and capital resources, risk assessment is incorporated into the decision-making process to guide primary care physicians in identifying those individuals who would most benefit from referral to nephrology services.

Currently, various international guidelines, including The Kidney Disease Improving Global Outcomes (KDIGO), the National Institute for Health Care Excellence (NICE) guideline and the Caring for Australians and New Zealanders with Kidney Impairment (CARI) guideline recommend an eGFR threshold of < 30 ml/min as a trigger for referral of individuals with CKD to nephrology services [23–25]. They also recommend referral based on urine albumin-to-creatinine ratio (ACR) thresholds as well as a rapid decline in eGFR. The 2021 revised NICE guideline and the Canadian Society of Nephrology have incorporated in their referral criteria a 5-year-risk of kidney failure (KF) of ≥ 5% and ≥ 3% respectively, using the 4-variable Kidney Failure Risk Equation (KFRE). Despite the availability of guidelines, the incidence of KF and the mortality associated with CKD continues to rise in some countries [26, 27], raising the possibility of poor implementation of preventative measures due to lack of adherence to guidelines by health care practitioners.

The objective of this literature review is to explore the risk assessment process and referral patterns of individuals with CKD from primary care to specialist nephrology services. It will focus on the primary-specialist care interface, the timeliness of referral, the adequacy of pre-dialysis care, and the role of clinical criteria vs. risk-based prediction tools in guiding the referral process.

## Method

### Introduction to review method

The increasing incidence and prevalence of CKD combined with evidence that timely and appropriate referral to nephrology services improves patient outcomes, whilst shortages in specialist nephrology services remain, has required system design improvements to streamline the referral process from primary to secondary care [13, 28–33]. Determining the appropriate time for referral has historically not incorporated risk-based assessment. Instead, the determination has largely been informed by studies examining timing in relation to the single outcome of progression to KF requiring kidney replacement therapy (KRT) [34]. This focus was reflected in the preliminary search conducted for this review, which found limited evidence around the timing for the broader spectrum of patients with CKD referred to nephrologists [35–38]. In addition, the KRT publications tended to describe the status of patients at the time of dialysis initiation relative to the time of referral, hence capturing the baseline characteristics for these individuals at the time of initiation of dialysis, rather than their first encounter with specialist nephrology services. Nevertheless, there was evidence showing benefit from early referral and appropriate pre-dialysis care for patients with progressive disease [13, 39–41]. However, the optimal timing of referral to nephrology services has not been established [42].

The preliminary search additionally found few studies that would meet the rigorous inclusion criteria for a meta-analysis or systematic review. To ensure the wide capture of literature examining the timing and referral for CKD management, a narrative review was chosen. Greenhalgh et al. [43] describe a narrative review as following a systematic process to produce an interpretive-qualitative report intended to explore, examine, and debate the current literature, i.e., it is not intended to address a specific research question. Accordingly, a narrative review is classified as a non-systematic review and does not use a risk bias tool for assessment [44] but can use a quality assessment tool if appropriate [45]. The studies were reviewed by two of the authors (CM and AR), who reached a consensus on the articles included in the review through collaboration and discussion.

### Search strategy and eligibility

In this review, the following key terms were identified and used to formulate a search strategy in PubMed: ‘chronic kidney disease’, ‘CKD’, ‘chronic renal failure’, ‘referral’, and ‘consultation’, ‘referr*’, ‘late referral’*, ‘early referral’*, ‘early nephrology referral’, ‘late nephrology referral’, ‘early nephrologist referral’, ‘late nephrologist referral’, ‘longer’, ‘nephrology care’, ‘pre-dialysis care’. The strategy included MeSH searches combined with keyword and synonyms text word searches. The following phrases were used to search in Google Scholar: ‘Primary care’, ‘specialty care interface in chronic kidney disease or CKD’, ‘timing of referral to nephrology or kidney clinic’, ‘referral patterns in chronic kidney disease and pre-dialysis care’. The search in Google Scholar was expanded to include ‘related articles’ and ‘cited by’ whenever an article of interest was identified. No restriction to the date of publication or age of the participants was applied and an experienced librarian assisted with the literature search strategies.

## Results

### Study selection

**Fig. 1:**
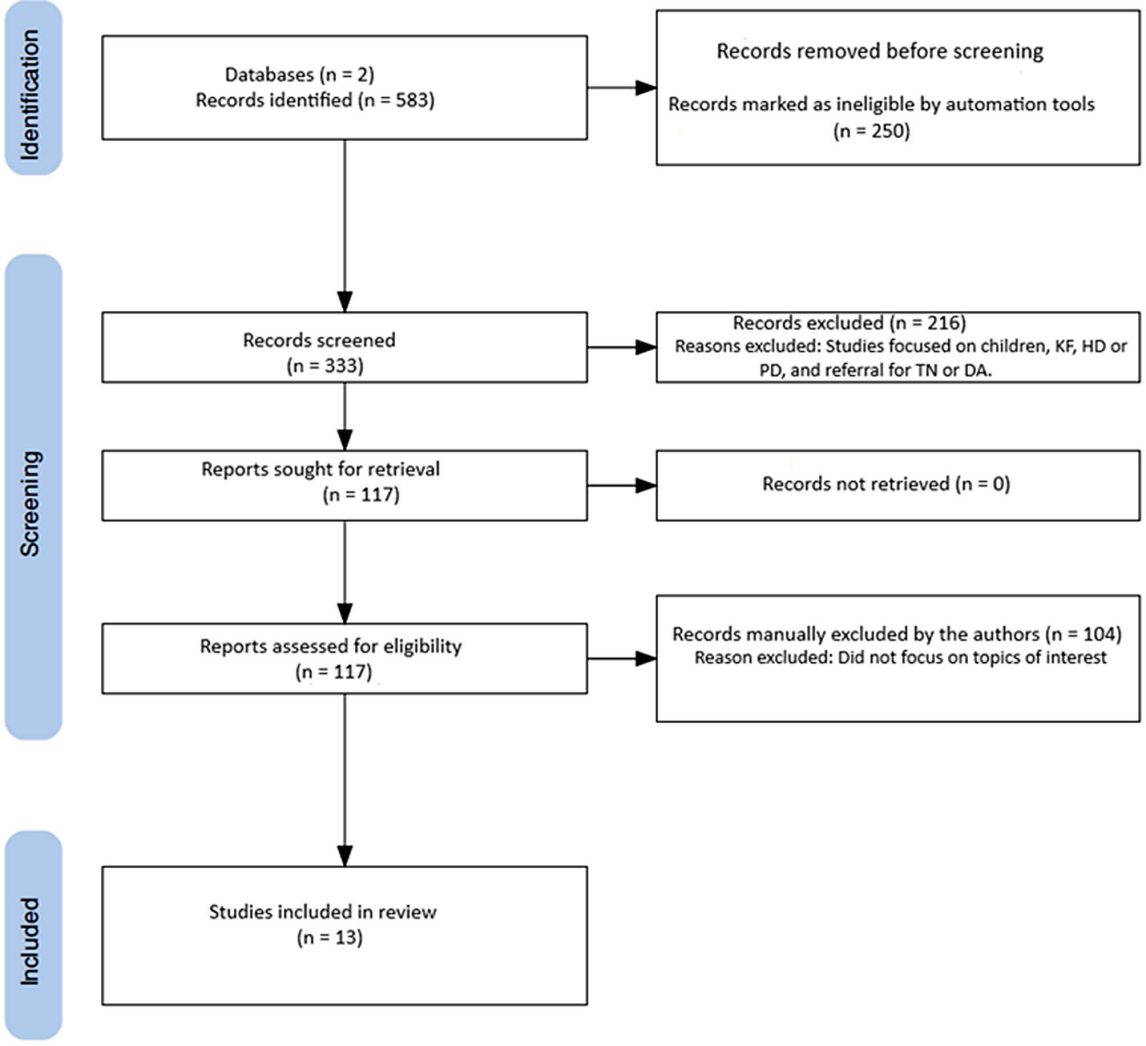
Flowchart of article selection on timing of referral to specialist services, adequacy of pre-dialysis preparation, and laboratory versus risk-based prediction tools. KF: kidney failure; TN: transplantation; DA: dialysis access; HD: haemodialysis; PD: peritoneal dialysis

Figure 1 shows that the PubMed and Google Scholar search returned 583 results with 117 remaining after automated filters, non-English articles with abstracts, case reports, editorial and comments, news and letters, books, reviews other than systematic reviews and meta-analysis, children, KF, haemodialysis (HD) or peritoneal dialysis (PD), and referral for transplantation or dialysis access were manually excluded after review of the abstracts. Of these, 13 studies were incorporated into the narrative review.

### Study characteristics

This paper summarises the literature on primary and specialist care interaction and referral patterns for individuals with CKD. Seven studies came from USA, two from the UK, and one paper each from Australia, South Korea, Canada, and France, with a total of 1,644,895 participants spanning the years from 1984 to 2021. The majority of the 13 studies were of retrospective design, one was a prospective observational study, one was a meta-analysis, one was a systematic review, and one was a cross-sectional population-based study.

### Assessment of study quality

We assessed the study quality using a modification of the US Preventive Services Task Force (USPSTF) criteria described by Fletcher et al. [46]. The USPSTF uses a hierarchy of overall study design as well as a rating of the internal validity, where a well-done randomized controlled trial constitutes the highest tier of evidence, while nonrandomized controlled trials, case-control studies, and cohort studies provide second-tier evidence. A three-category rating has also been added to distinguish among good, fair, and poor, to evaluate the internal validity of different study designs: systematic reviews, case–control studies, RCTs, cohort studies [47]. Generally, a good study meets all criteria for that study design, while a fair study does not meet all criteria but does not contain enough flaws that invalidate its findings and a poor study is considered to have enough flaws to invalidate its results. We rated the systematic review and two of the retrospective studies as being of good quality since they met all criteria for their study designs, with the remaining studies identified as being of fair quality.

We grouped the results into three main themes as follows:

1. Optimal timing of referral to nephrology services (Table 1).
2. Adequacy of preparation for kidney replacement therapy (Table 2).
3. Clinical criteria vs. risk-based prediction tools (Table 3).

**Table 1:** Studies evaluated timing of referral to nephrology services with hospitalization and/or mortality as the outcomes

Paper number	Author(s)	Population	Study design and Quality Rating	Characteristics of Participants	Definition of late referral	Follow-up	Comments and results
1	Chan et al. 2007 [36]	22 studies (12,749 participants)	Meta-analysis QR = Fair	Studies with defined timing of referral and assessment of outcomes related to mortality or duration of hospitalization	1 to 12 months	NA	• 20 studies (total *n* = 12,018) that presented mortality data in early and late referred groups of subjects found that Late nephrology referral of CKD patients was associated with a significantly increased risk of death (relative risk [RR] 1.99; 95% CI [1.66, 2.39]; *P <* .0001) • 8 studies that assessed timing of referral and its impact on duration of hospital stay (total *n* = 3220) at the time of initiation of KRT found that late referred patients had a prolonged duration of hospitalization compared to the early referred patients by an average of 12 days
2	Stroupe et al. (2011) [48]	8,022	Retrospective study QR = Good	Kidney failure commencing KRT	< 3 months	1 year	• 37% received no pre-dialysis nephrology care, 24% received low, 16% moderate, and 23% high-intensity pre-dialysis nephrology. • During the year after dialysis initiation, patients in these groups spent an average of 52, 40, 31, and 27 days in the hospital (*P* < .001), respectively. • Greater intensity of pre-dialysis nephrology care was associated with lower costs even among patients whose first pre-dialysis nephrology visit was ≤ 3 months before dialysis initiation. • Patients with greater pre-dialysis nephrology care had lower mortality rates during the year after dialysis initiation (43%, 38%, 28%, and 25%, respectively, *P* < .001).
3	Kim et al. 2013 [49]	1,028	Prospective cohort study QR = Fair	Patients aged > 20 years starting dialysis for kidney failure	< 12 months	2 years	• Time from referral to dialysis was significantly longer in 599 ER patients than in 429 LR patients (62.3 ± 58.9 versus 2.9 ± 3.4 months, *P* < .001). • Emergency HD using a temporary vascular catheter was required in 485 (47.2%) out of all patients and in 262 (43.7%) of ER compared with 223 (52.0%) of LR (*P* = .009). • After 2 years of follow-up, the survival rate in ER was better than that in LR (HR 2.38, 95% CI [1.27, 4.45, *P* = .007]).
4	Smart et al. 2014 [50]	40 studies (63,887 participants)	Systematic review QR = Good	Studies of adult patients with CKD receiving dialysis with a known date on when they were initially referred to a specialist nephrology service	1 to 12 months	NA	• Compared to patients referred late, mortality was reduced across three, six, 12 and 60 months in those referred early • 4 studies reported mortality at three to four months data pooling showed mortality was reduced in patients referred early by 39% • One reported mortality at six months which was 42% lower in those referred early. • 16 studies reported 12-month mortality which was 35% lower in those referred early. • 3 studies reported five-year mortality data pooling showed mortality reduction of 33% in those referred early
5	Gillespie et al. 2015 [51]	443, 761	Retrospective cohort study QR = Fair	Patients starting dialysis or receiving a kidney transplant	< 12 months	1 year	• Overall, 33% of new KF patients had received no nephrology care, 14% had received < 6 months, 25% had received ≥ 6 but ≤ 12 months and 28% had received > 12 months of care prior to KF onset. • Any nephrology care prior to KF onset (versus no care) was associated with better health status and preparedness at the start of KF. • Longer pre-KF nephrology care was associated with lower first-year mortality (adjusted hazard ratio = 0.58 for > 12 months versus no care; 95% CI [0.57, 0.59]), higher albumin and haemoglobin, choice of peritoneal dialysis and native fistula and discussion of transplantation options.

ER: Early referred; LR: Late referred; KRT: Kidney replacement therapy; KF: Kidney failure; HD: Haemodialysis; QR: quality rating

**Table 2:** Studies evaluating adequacy of preparation for kidney replacement therapy with timing of referral, quality of pre-dialysis care, intensity of pre-dialysis care as the outcomes

Paper number	Authors(s)	Population	Study design and Quality Rating	Characteristics of Participants and Methods	Exposure of interest	Follow-up	Comments and results
6	Avorn et al. 2002 [52]	3,014	Retrospective study QR = Fair	Patients who underwent maintenance dialysis between 1991 to 1996	Timing of nephrology referral & Frequency of nephrologist care	1 year	• 1430 (47.4%) died in the first year of dialysis • 1288 (42.7%) were between the ages of 65 and 74 years and 1063 (35.3%) aged 75 to 84 years • Approximately one third of patients (34.5%) were referred late (did not see a nephrologist until 90 days or less before their first renal replacement therapy • Half of the patients (50.5%) had fewer than 5 nephrologist consultations in the year prior to renal replacement therapy. • Patients with late referral had a 37% increase in risk of death in the first year of dialysis compared with patients referred early (95% CI [0.22, 0.52]; *P* < .001) • Patients who saw a nephrologist on fewer than 5 occasions in the year prior to dialysis had a 15% higher mortality rate in the first year of dialysis compared with those who had had 5 or more nephrologist visits (95% CI [1.03, 1.28]; *P* = .01).
7	Thilly et al. 2012 [53]	566	Observational cohort study QR = Fair	Adult patients with chronic kidney disease starting dialysis in Lorraine (France) between 2005 and 2006	Quality of pre-dialysis care	1 year	• Overall, 142 patients (25.1%) died during the first year of KRT. • Quality of pre-dialysis therapeutic practices was high in 18.2% of the 566 included patients, moderate in 62.5%, and poor in 19.3%. • Crude survival rates at 1 year were 82.5% for patients who had received high quality of care, 74.6% for those receiving moderate quality of care, and 68.5% in the poor group. • Quality of pre-ESKD therapeutic practices was significantly associated with survival: the higher the quality, the better the survival (high: HR 1; moderate: HR 1.56, 95% CI [0.93, 2.60]; poor: HR 1.95, 95% CI [1.08, 3.50]). • Quality of therapeutic practices was not associated with duration of hospitalization among the 390 surviving patients at 1 year.
8	Foley et al. 2014 [57]	498,566	Retrospective study QR = Fair	Adults aged ≥ 18 years who initiated dialysis between 2005 and 2009 in the USA	Duration of pre-dialysis care	1 year	• Mortality rates were initially unexpectedly low (week 1), peaked at 37.0 per 100 person-years in week 6, and declined steadily to 14.8 by week 51. • Early mortality rates were particularly high in older subgroups, in patients whose duration of pre-dialysis nephrologist care was short, and in hemodialysis patients, especially those with catheters for vascular access.
9	Singhal et al. 2014 [56]	12,143	Retrospective cohort study QR = Fair	Adults with prior outpatient nephrology care who started kidney replacement therapy (KRT) for end-stage renal disease (ESRD) between 1998 and 2008.	Intensity of pre-dialysis care	1 year	• 75.9% of the 12,143 patients had early CKD care (starting KRT ≥ 6 months after first outpatient nephrology visit). • Only 38.3% of the early group had high cumulative (> 10 visits) and consistent critical period care (seen in ≥ 3 of the 6 months) prior to start of RRT. • One year after start of RRT, 15.8% of the population had died. • A 1-year mortality was more likely with late care, lower cumulative care, and inconsistent critical period care. • Late CKD care (< 6 months) predicted increased mortality 1 year after start of KRT (adjusted odds ratio or AOR = 1.31 (95% CI [1.13, 1.53]). • A graded survival benefit was seen with cumulative visits (AOR 6–10:1–5 visits = 0.75 (95% CI [0.64, 0.88]; AOR = > 10:1–5 visits = 0.68 (95% CI [0.57, 0.81])) • Reduced mortality was seen with consistent critical period care (AOR = 0.73; 95% CI [0.64, 0.82]). • The beneficial effect of CKD care on early survival relied not only on timely referral but also on the total number of visits during the follow-up and the number of visits during the last 6 months before dialysis. • Although an early referral seems to be necessary, it is not sufficient to define optimal pre-dialysis renal care, which also includes quantity and consistency of care
10	Fischer et al. 2016 [58]	58,014	Retrospective cohort study QR = Good	Elderly patients (≥ 66 years old) who initiated dialysis for ESKD between January 1, 2000, and December 31, 2001	Intensity of pre-dialysis care	2 years	• Overall, 46% had no pre-dialysis nephrology care, 22% had low intensity care (1–3 visits), 13% had moderate intensity care (4–6 visits), and 19% had high intensity care (> 6 visits). • Patients who received a greater intensity of pre-dialysis nephrology care had a higher prevalence of permanent vascular access (both fistula and graft) and a lower prevalence of severe anaemia and very low eGFR at the time of dialysis initiation. • Use of PD within 60 days of dialysis initiation was more frequent in patients with greater intensity of pre-dialysis nephrology care (*p* < .001). • The percentage of patients who died within 2 years of dialysis initiation was 59.7% (15,991/26,789), 55.0% (6,916/12,566), 48.0% (3,694/7,690), and 42.7% (4,689/10,960) among those who received no, low intensity, moderate, or high intensity pre-dialysis nephrology care, respectively (*p* < .001) • The percentage of patients who received a kidney transplant within 2 years of dialysis initiation was 0.4% (106/26,798), 0.6% (76/12,566), 1.0% (75/7,690), and 1.5% (164/10,960) among patients who received no, low intensity, moderate, or high intensity pre-dialysis nephrology care, respectively, (*p* < .001). • Survival following dialysis initiation was longer among patients with a greater intensity of pre-dialysis nephrology care, with median days of survival of 502, 618, 730, and 730 among patients who received no, low intensity, moderate, or high intensity nephrology care in the pre-dialysis period, respectively (*p* < .001) • Results were similar in subgroup analyses among patients with late nephrology care (first nephrology visit < 3 months before dialysis initiation) among patients ≥ 75 years. • The risk of permanent vascular access at dialysis initiation was 57% greater with low intensity (RR = 1.57, 99% CI [1.48, 1.67]), 161% greater with moderate intensity (RR = 2.61, 99% CI [2.45, 2.77]), and 260% greater with high intensity pre-dialysis nephrology care (RR = 3.60, 99% CI [3.42, 3.794]). • The risk of death within 2 years after dialysis initiation was 6% lower among patients with low intensity (RR = 0.94, 99% CI [0.92, 0.97]), 13% lower for patients with moderate intensity (RR = 0.87, 99% CI [0.84, 0.91]), and 20% lower for patients with high intensity pre-dialysis nephrology care (RR = 0.80, 99% CI [0.77, 0.82]).

AOR: Adjusted odds ratio; AHR: Adjusted hazard ratio; KRT: Kidney replacement therapy; PNV: pre-nephrology visit; MNC: month of nephrology care; PD: Peritoneal dialysis; QR: quality rating

**Table 3:** Studies comparing clinical criteria vs. risk-based prediction tools

Paper number	Author(s)	Population	Study design and Quality Rating	Characteristics of Participants and Methods	Follow-up	Comments and results
11	Major et al. 2019 [59]	35,539	Primary care Retrospective cohort QR = Fair	Primary care patients with CKD who attended GP practices	2 years	• Using the recalibrated KFRE alone led to either (1) no improvement in the number of cases who went on to KF who were not referred or (2) large increases in the proportion of the CKD population referred, compared to the current NICE recommendations. • A hybrid approach of referral using a KFRE criterion 5-year KF risk ≥ 5% and ACR ≥ 70 mg/mmol compared to 2014 NICE criteria, led to additional KF cases being detected, whilst also decreasing potential referrals to secondary care.
12	Duggal et al. 2021 [60]	399,644	Observational cohort study QR = Fair	Chronic kidney disease Number of patients identified for referral	1 year	• Among 362,084 patients who had not previously seen a nephrologist, 66,276 (18.3%) met an indication for referral, and 11,752 (17.7%) were referred. • A total of 295,808 patients did not meet a referral indication, and 10,015 (3.4%) were referred. • Most patients who met an indication for referral did so based on the eGFR < 30 mL/min/1.73m^2^ criterion. • If a minimum kidney failure risk of 1% over 2 years were applied to all new patients meeting laboratory-based potential referral indications, the number of patients targeted for nephrology referral would be reduced from 66,276 to an estimated 38,229 patients, a 42.3% reduction. • Referral based only on predicted risk would result in a similar number of patients identified for referral at a predicted risk threshold of 2% or higher. • The application of more stringent risk thresholds would identify progressively fewer patients. • Targeting referrals based on the KFRE could result in fewer referrals of patients with rapid eGFR decline. • It may not currently be feasible to care for all patients who meet guideline-suggested laboratory-based indications for nephrology referral.
13	Bhachu et al. 2021 [61]	107,962	Cross-sectional population-based observational study. QR = Fair	Patients with CKD stage G3-5	15 months	• Using a risk-based threshold for patients with CKD rather than the current NICE CKD guideline criteria would lead to a major change in referral patterns of individuals from primary to specialist care. • Approximately 40% of patients with a > 3% risk of ESRD by 5 years are missed by the current NICE referral criteria. • Approximately one-third of patients who fulfill the current NICE criteria are at low risk of ESKD (KF) (≥ 3% at 5-years) including more than half of those with a sustained decrease in eGFR as defined in the NICE guidance. • Some patients at low risk of progression to KF are accessing limited specialist nephrology resources, whereas others with a higher risk of progression do not meet the NICE criteria and are not identified as requiring referral. • If KFRE rather than NICE CKD criteria was used for referral of patients, just under 15% of patients with CKD would be reclassified between primary and specialty care.

*KFRE: Kidney failure risk equation; eGFR: Estimated glomerular filtration rate; CKD: Chronic kidney disease; NICE: The National Institute for Health and Care Excellence; KF: Kidney failure; QR: quality rating*

### Theme 1: Optimal timing of referral to nephrology services

Table 1 shows that five studies explored the timing of referral with hospitalization and/or mortality as the outcomes (reference numbers 36, 48–51).

Chan et al. [36] conducted a meta-analysis of 22 studies comprising 12,749 participants to compare differences in mortality and the duration of hospitalization in patients with CKD who were referred early versus late to nephrologists. The studies that compared mortality in early and late referred groups found that late nephrology referral of CKD patients was associated with a significantly increased risk of death, whereas the studies that assessed timing of referral and its impact on duration of hospital stay at the time of initiation of KRT found that late referred patients had a prolonged duration of hospitalization compared to the early referred patients by an average additional 12 days. The authors recommended that greater attention should be directed at increased education of primary care providers and patients on CKD care and the value of co-management and timely referral.

Using a cohort of elderly patients who commenced dialysis, Stroupe et al. [48] conducted a retrospective study to compare healthcare costs for patients who received different levels of predialysis care during the year before dialysis initiation with those who received no predialysis care. They found that greater intensity of predialysis nephrology care was associated with lower costs even among patients whose first predialysis nephrology visit was ≤ 3 months before dialysis initiation and that patients with greater predialysis nephrology care had lower mortality rates during the year after dialysis initiation.

In a multi-centre, prospective cohort study of patients who were initiated on dialysis therapy, Kim et al. [49] explored the impact of early nephrology referral and frequent attendance at nephrology clinics before KRT initiation, on patient survival. The likelihood of patients referred early (ER) receiving emergency HD using a temporary vascular catheter was significantly reduced compared to those referred late (LR) (43.7% vs. 52.0%), and that the 2-year survival rate in ER was better than that in LR.

Smart et al. [50] conducted a systematic review of 40 longitudinal cohort studies on 63,887 participants to evaluate the benefits and harms of early versus late referral to specialist nephrology services in CKD patients who are progressing to KF and KRT. Using more than 6 months as the interval between first nephrology evaluation and start of dialysis to define early referral, they found that 68% of participants were referred early. Reduced mortality and hospitalisation, better uptake of PD and earlier placement of arteriovenous fistulae for patients with CKD was observed in those participants who were referred early to a nephrologist. They concluded that their findings aligned well with previously published systematic reviews on the topic, showing unequivocal benefits of early referral.

By analysing nationwide US KF data from 2006 to 2010 to explore variations in predialysis nephrology care among incident dialysis patients, Gillespie et al. [51] investigated whether longer predialysis nephrology care was associated with lower mortality at, and following, the onset of dialysis. Their analysis showed that any nephrology care prior to KF onset (versus no care) was associated with better health status and preparedness at the start of KF. They also determined that longer predialysis nephrology care was associated with lower first-year mortality, higher albumin and haemoglobin, choice of PD and native fistula and discussion of transplantation options. Early referral of CKD patients may, if the disease progresses to KF, reduce first-year mortality after dialysis onset, presumably by improving the patient’s health and readiness for KRT.

### Theme 2: Adequate preparation for kidney replacement therapy

Table 2 shows that in this review, five papers explored the adequacy of predialysis care with timing of referral, quality of pre-dialysis care, intensity of pre-dialysis care as the outcomes, four of which were retrospective, and one was a prospective study [52, 53, 56–58]. Avorn et al. [52] analysed data describing all health care encounters for patients with KF between January 1991 and June 1996 to determine whether late referral to a nephrologist in patients with CKD influenced the adequacy of vascular access for HD. Their study demonstrated that approximately one third of patients were referred late (did not see a nephrologist until 90 days or less before their first KRT), and half of the patients had fewer than 5 nephrologist consultations in the year prior to KRT. Patients with late referral had a 37% increase in risk of death in the first year of dialysis compared with patients referred early, and patients who saw a nephrologist on fewer than 5 occasions in the year prior to dialysis had a 15% higher mortality rate in the first year of dialysis compared with those who had had 5 or more nephrologist visits.

Thilly et al. [53] examined the association between quality of predialysis care and either survival or hospitalisation during the first year of dialysis. They found that quality of predialysis therapeutic practices was significantly associated with survival, with higher quality portending better patient survival. However, they found no association between quality of therapeutic practices with duration of hospitalization among those patients who survived for at least one year, a finding that they attributed to the lack of power in their small sample size.

Further large cohort studies of adult patients, including older adults treated with chronic dialysis, have demonstrated that greater intensity of predialysis nephrology care is associated with more favourable outcomes [54, 55]. For example, Singhal et al. [56] used the concept of cumulative and consistency of care in referred patients to predict survival and other important outcomes in the incident KRT population, independent of the traditional measure of early versus late care. They studied a total of 12,143 adults with prior outpatient nephrology care who started HD or PD or received a kidney transplant in Ontario between 1 July 1998 and 31 March 2008 and examined the relationship between alternate measures of CKD care to mortality and other outcomes. Their findings were quite interesting in that patients traditionally classified as receiving early CKD care often did not receive adequate care immediately prior to initiating KRT. Rather, alternate measures of CKD care such as cumulative care and consistent critical period care were independent predictors of survival and other important outcomes in the incident KRT population, independent of the traditional measure of early versus late care. For example, their study showed that cumulative CKD visits and visits in at least 3 of the 6 months prior to start of KRT predicted reduced 1-year mortality, reduced inpatient start of KRT, and increased predialysis vascular access creation. One-year mortality was more likely with late care, lower cumulative care, and inconsistent critical period care. Cumulative care and consistency of care during the critical period before commencement of KRT predicted mortality and other secondary outcomes, independent of the traditional measure of early versus late care.

Foley et al. [57] conducted a retrospective study of US adult patients who commenced dialysis between 2005 and 2009 and evaluated the weekly mortality rates during the first year of KRT (early and later mortality) and determined the week at which peak mortality rates occurred. They demonstrated that the highest mortality occurred at week 6, and that early mortality rates were particularly high in older subgroups, in patients whose duration of predialysis nephrologist care was short, and in HD patients, especially those with catheters for vascular access.

Fischer et al. [58] performed a retrospective study on a cohort of older patients, aged ≥ 66 years, who commenced dialysis between 2000 and 2001. They evaluated the relationship between predialysis nephrology care and a range of dialysis-related clinical outcomes, including the relationship between frequency of predialysis nephrology visits and outcomes at dialysis initiation and health outcomes after initiation. Patients with a greater intensity of predialysis nephrology care had more favourable health parameters and outcomes at the time of dialysis initiation and for the first two years following initiation. Greater intensity of predialysis nephrology care was also associated with a higher prevalence of permanent vascular access and a lower prevalence of severe anaemia and very low eGFR at the time of dialysis initiation. Use of PD within 60 days of dialysis initiation was more frequent in patients with greater intensity of predialysis nephrology care. A higher number of predialysis visits was associated with decreased risk of death and higher chance of kidney transplantation during follow up.

### Theme 3: Clinical criteria versus risk-based assessment tools

Table 3 details the three papers reviewed that considered the question of clinical criteria versus risk-based assessment tools [55–57]. Major et al. [59] compared the implementation of a combination of the NICE referral guidelines and the 5-year 4-variable KFRE risk of ≥ 5% of KF using a UK primary care cohort. They found that using the recalibrated KFRE alone led to no improvement from the current NICE recommendations. However, using the hybrid model of a ≥ 5% risk of KF over 5 years from the recalibrated KFRE and/or a urine ACR of ≥ 70 mg/mmol would reduce the number of individuals eligible for referral without increasing the number who later develop KF and are not initially eligible for referral. Their findings therefore suggested that the hybrid model appeared to identify patients in whom the risk of KF was better than the guideline alone, whilst decreasing nephrology referrals. They add that use of the KFRE-based hybrid model may lead to more appropriate referrals to secondary care, with the overall impact of large cost savings across their healthcare system.

Duggal et al. [60] conducted a retrospective study on 399,644 individuals with CKD and evaluated whether nephrology referral patterns followed current clinical practice guidelines and how utilising risk-based thresholds would impact the volume of referrals. They found that among 362,084 patients who had not previously seen a nephrologist, 18.3% were eligible for referral according to the guidelines, and of these, 17.7% were referred. Of the 295,808 who did not meet a referral indication 3.4% were referred. Laboratory-based criteria and KFRE risk thresholds identified similar numbers of patients for referral, although the patients identified using the 2-year KFRE had a higher median risk of KF than those patients who fulfilled the laboratory criteria (2.3% vs. 1.5%). In addition, combining the laboratory-based criteria with 2-year KFRE risk of ≥ 1% would reduce eligible referral volume by 42%. Their findings suggested that the KFRE is more effective at identifying those patients who are at a higher risk, who are most in need of specialised care.

Bhachu et al. [61] used a cross-sectional population based observational study to compare the NICE 2014 CKD guidelines and the 4-variable KFRE risk of > 3% of KF at 5 years on patients identified with CKD stage G3-5 in United Kingdom primary care registered in The Health Improvement Network database. They found that approximately one-third of patients who fulfill the current NICE criteria are at low risk of KF, including more than half of those with a sustained decrease in eGFR as defined in the NICE guidelines. In addition, some patients at low risk of progression to KF were found to be accessing limited specialist nephrology resources, whereas others with a higher risk of progression do not meet the NICE criteria and are not identified as requiring referral. Applying the 5-year KFRE threshold of > 3% would yield similar eligible referral volume as those identified by NICE criteria, but the later would miss 40% of patients with KFRE risk of > 3%. The authors concluded that a risk-based referral approach would lead to a major change in referral patterns of individuals from primary to specialist care and a substantial reallocation of patients between primary care and specialist nephrology care with only a small increase in numbers eligible, ensuring those at higher risk of progression are identified.

## Discussion

This narrative review examined 13 papers to explore the risk assessment process and referral patterns of individuals with CKD from primary care to specialist nephrology services with a focus on the primary-specialist care interface, the timeliness of referral, the adequacy of pre-dialysis care, and the role of clinical criteria vs. risk-based prediction tools in guiding the referral process. The findings were grouped into three themes, which are discussed in turn.

### Optimal timing of referral to nephrology services

The World Health Organization [62] promotes the establishment of successful and collaborative relationships between primary and specialist care and advocates effective communication as the cornerstone for the successful interface. In the management of CKD, this collaboration needs to be conducted in a timely manner to ensure optimal care of individuals with the disease, the marker of which is early detection and timely intervention, including early referral of those with need of nephrology care. An effective referral process underpins this relationship by ensuring the provision of optimal care at the appropriate level, and cost-effective utilisation of resources, delivered in a timely manner (early referral). This venture can be further enhanced by the development of integrative care models between primary care and secondary care, aided by incorporation of risk prediction tools to trigger the timing of referral, with a focus on reducing unnecessary workload for secondary care resources whilst upholding a high standard of care delivered to the patient.

The reviewed literature demonstrated the unequivocal benefit of early referral of individuals with CKD to nephrology services, irrespective of the definition of “early referral” used, in the management of both dialysis-related and non-dialysis related issues. However, despite the overwhelming evidence supporting early referral, the literature still shows that up to a third of CKD patients are still referred late [35, 40, 63, 64].

There are some disadvantages of using the timing of referral in relation to the commencement of dialysis to define “timeliness of referral”. Firstly, it will only capture those individuals who have survived to initiation of KRT, thus using this definition will also miss individuals who were referred late in the course of their CKD but died prior to KRT start. The latter cohort are likely to differ in their baseline characteristics from those who survive to initiate KRT. In addition, since only a fraction of individuals with an eGFR < 30 mL/min go on to require KRT [3], studies that rely on this retrospective measure to define early referral would also not examine the volume of patients who might have been followed and managed, possibly quite appropriately, for progressive CKD without requirement for KRT. Moreover, the other important goals of early referral such as providing specific therapy based on an accurate diagnosis, slowing CKD progression, managing of comorbid conditions including cardiovascular disease (CVD), as well as identifying and managing CKD-specific complications will inadvertently be relegated to secondary goals.

Over the years, there has been greater appreciation of the epidemiology of CKD where it has become apparent to clinicians that the major competing risk for dialysis is death from CVD [65, 66]. This has resulted in broadening the focus of CKD care to include CVD risk reduction, in addition to or concomitant with, reducing the progressive decline in kidney function. Pursuant to this has been the proliferation of nephrology literature highlighting the importance of early detection and timely intervention and prevention of CKD and the need for mitigating its impact on society [42, 67–69]. Black et al. [70] emphasize one of the goals of the 2008 NICE guidelines, which was to strike a balance between early referral and service capacity whilst identifying uncertainties around the potential benefits (and harms) of early referral. Hence, in addition to affording the patient a planned start to KRT, earlier referral also offers the opportunity to intervene to delay progression of kidney disease by offering appropriate use of kidney-protective interventions and treat its complications at this early stage, and to prevent CVD by allowing appropriate use of cardioprotective interventions.

Notwithstanding this skewed focus on advanced CKD, there is ample evidence from Registry data and findings from many publications of a significant increase in the burden of CVD and death in individuals with eGFR of less than 60 ml/min (but above the recommended cut-off for referral of 30 ml/min), many of whom would also benefit from specialised care offered in multidisciplinary CKD clinics [70]. The challenge therefore lies in identifying not only those individuals who are at risk of progressing to KF, but also those who are at increased risk of developing CVD or the outcome of premature death. This is further underscored by the impossible proposition of referring all individuals with CKD G3 (eGFR between 30 and 60 ml/min) to the nephrologist as this would overwhelm the limited nephrology workforce. Identifying those individuals who are likely to benefit from specialised nephrology care would promote the appropriate use of limited workforce resources and avoiding inundation of specialist CKD clinics.

### Adequacy of preparation for kidney replacement therapy

The adequacy of predialysis care has received increasing attention in recent times. It has traditionally been described in relation to the duration of nephrology care before an individual commences KRT [34, 71, 72]. The same time frame is used to distinguish early referral from late referral to nephrology care and the subsequent comparison of outcomes after commencing KRT [73–76]. This definition is naturally flawed as it doesn’t consider the type or quality of care that the individual receives during the specified period before they commence KRT. It also doesn’t consider that quickly deteriorating patients will have had less opportunity for previous care, hence raising the question whether the late referral is the cause of the poor outcomes or the “poor outcomes” (rapid deterioration) the cause of the late referral (early dialysis initiation).

Obrador and Pereira offer a comprehensive enunciation of what constitutes optimal predialysis care [77]. They identify early interventions aimed at slowing down of CKD, prudent management of CKD complications, timely referral to allow adequate preparation for KRT, and exposure to multidisciplinary educational programs as components of adequate predialysis care. In reference to early interventions, the benefits accrued from renin angiotensin aldosterone system (RAAS) inhibitors, glycaemic control and lipid lowering therapies during the course of CKD may extend to reduction of comorbid complications of CKD such as CVD and hence ameliorating the associated high cardiovascular morbidity and mortality rates [78–80]. Similarly, adequate preparation for KRT entails the provision of the opportunity for exposure to multidisciplinary educational programs and psychosocial counselling geared towards appropriate modality selection, and timely placement of access for dialysis [81, 82]. Adequate preparation should also allow for the correction of uraemic complications such as hypoalbuminemia, anaemia, acidosis, and CKD mineral and bone disorder (CKD-MBD), which have all been associated with improved outcomes after commencement of dialysis [13].

Researchers have recently started to look beyond the interval between the first visit to the nephrologist and the commencement of KRT as the sole determinant of the adequacy of predialysis nephrology care. This was mostly prompted by the persistence of high rates of suboptimal KRT starts and mortality even in patients referred early to nephrology services [83–85].

Commencing KRT with permanent vascular access has also been used to quantify the adequacy of predialysis care. The fistula first policy is borne out by strong evidence that the arteriovenous fistula (AVF) is the preferred vascular access for HD owing to the lower rates of infection and mortality and good long-term patency [86–88]. Late referral to nephrologists has been singled out as one of the frequent reasons for the high rate of use of catheters and underutilisation of fistulae and grafts, making the type of access at the initiation of KRT one of the indicators of adequacy of predialysis care [89].

Adequate predialysis care should therefore encompass the amount and quality of care afforded to the patient in the early stages of CKD when interventions to prevent or attenuate progression to KF and development of CVD have got the greatest chance of making an impact, in addition to optimising care in preparation for KRT in those with advanced stages of their disease. Hence, although an early referral seems to be necessary, it is not sufficient to define optimal predialysis nephrology care, which also includes quality, quantity, and consistency of care.

### Reconciling timing of referral and adequacy of dialysis care

It is important to the practising clinician that a distinction is made between timing of referral, a concept that is time-based, and adequacy of pre-dialysis care, which includes duration of pre-dialysis care but also incorporates the intensity/frequency and quality of care. Chan et al. [36] highlight the importance of this distinction in their meta-analysis by proposing an alternate approach to the concept of early referral, where it might be helpful to identify the specific interventions that may contribute to the positive impact of early referral once patients commence KRT. For example, some studies in their meta-analysis showed that the benefits of early referral, such as lower mortality after commencement of KRT, could be attributed to the early referred patients having lesser degree of anaemia, higher pre-dialysis albumin, and better pre-dialysis BP control. In particular, one paper in their analysis found that patients who received more consistent pre-KRT epoetin treatment had higher haematocrits before the start of KRT, and lower risk of death 1 year after the 1st dialysis session [90]. While acknowledging that the patients who received less consistent epoetin may have been more likely to have LVH at the initiation of KRT and hence have a higher risk of death once on dialysis, they also considered the possibility that frequency of epoetin treatment may also have been associated with increased exposure to the healthcare system, and hence allowing patients to see the healthcare professionals more frequently.

While it can be argued that timing of referral alone (i.e., duration of pre-dialysis care) cannot be sufficient to account for the superior outcomes observed in early referred patients, it is also instructive to acknowledge that a longer duration of pre-dialysis care creates opportunities for an increased number of visits to the nephrology clinics and allows for more time for effective treatment and correction of anaemia, hence lowering the risk of death, and additionally, provides more time for successful establishment of permanent vascular access which is known to improve outcomes on dialysis. Avorn et al. [52] suggest that seeing a nephrologist earlier and more frequently allows for better management of comorbid conditions which impact the progression of CKD, including hypertension, CVD, and CKD-MBD, while creating opportunities for multidisciplinary input, which in turn may prepare the patient better for KRT. Better pre-dialysis preparation is likely to promote compliance with dietary and fluid restrictions on dialysis, hence mitigating the risk of poor outcomes.

This point is emphasised by Foley et al. [57], who concluded that in order to optimise outcomes, it is not only the timing of referral that is important, but also the frequency and regularity with which patients are seen, which in turn allows for timely interventions, such as planning for access. In their commentary on Singhal and Foley’s [56, 57] papers, Rognant and Laville [91] observe that although early referral might be necessary (i.e., timely referral), it is not sufficient to define optimal pre-dialysis care, which should also consider the quantity and consistency of the care delivered. The duration, the quantity, and the consistency of care should therefore characterise the multifaceted and comprehensive definition of adequacy of pre-dialysis care.

Appreciation of this distinction opens opportunities for future research to explore the determinants of adequate pre-dialysis care, which should include the roles of various multidisciplinary professionals, such as primary care physicians, the nephrologist, dieticians, clinical psychologists, vascular access coordinators, and nurse practitioners, in the quest to deliver optimal care to the patient, that can mitigate unfavourable outcomes which are so prevalent after initiation of dialysis.

### Clinical criteria vs. risk-based prediction tools

Evidence demonstrated that recommending referral of individuals with CKD from primary to secondary care based on laboratory criteria via practice guidelines rather than future risk, may contribute to referral of individuals who could be considered ‘low risk’. This may lead to an increase in inappropriate referrals, and consequently to an increase in the workload and cost of caring for CKD patients with little apparent benefit [92, 93]. The current thresholds for the trigger to refer to the nephrology clinic include an eGFR of < 30 ml/min (uncalibrated for age or sex), severe albuminuria or proteinuria, or an eGFR decrease > 5 mL/min per year. The rationale behind the cut-off point of < 30 ml/min presupposes that once the eGFR drops to less than 30 ml/min (stage G4 CKD), the risk of progression to KF becomes significant. However, it is also seen that many patients with stage G4 CKD do not progress to KRT. For example, Ravani et al. [65] conducted a population-based cohort study of approximately 4 million people to investigate the risks of KF and death in adults with incident stage G4 CKD. Of the 30,801 adults who developed stage G4 CKD, they found that on average, death was 3 times more likely to occur than KF, 6 times more likely than KF among those aged 75 to 84 years, and 25 times more likely than KF among those aged 85 years or older. Furthermore, evidence suggests that risk exists in both individuals with stage G4 CKD as well as those with stage G3 CKD, whereby people within the same CKD classification can have divergent absolute risks, with substantial overlap existing between different categories [94, 95]. As such, timely intervention in both early and late stages of CKD is likely to mitigate both disease progression as well as the threat of CVD [60]. Some scholars have argued that focusing on referral at eGFR < 30 ml/min limits the impact of intervention as the damage to the kidney is already advanced, minimising the opportunities for slowing progression [96].

It is therefore logical to contend that the timing of referral should be guided by the risk of progression to KF or the risk for development of CV events or death, which can be estimated by making use of prediction models. As demonstrated in this review, development and application of prediction models such as the KFRE has led to incorporation of risk prediction into some current guidelines. The updated NICE guidelines integrated the KFRE in 2021, with the recommendation to use a threshold of > 5% over a 5-year period as one of the triggers for referral [97]. Several provinces in Canada have adopted the use of the KFRE as one of the criteria to guide the decision of referral to nephrology services, a position that has also been embraced by the Kaiser Permanente health system in the United States [98].With the use of such models, most patients with stage G3 CKD can be stratified as low risk and can potentially be treated solely by their primary care provider, whereas those at high risk can be referred for urgent care by a nephrologist [60, 61].

### Limitations

Our review has got several limitations. A major limitation is the paucity of prospective data/RCTs given that the vast majority of studies were retrospective. Importantly, the reviewed literature showed a lack of consensus on the definition of early or late referral. Furthermore, most of the literature describes data for individuals commencing dialysis (early or late referrals) rather than a broader spectrum of all patients with CKD under nephrology care. Three or four months is the most used cut-off for defining late referral in older studies, whereas 6 or 12 months is more commonly used in recent studies. The differing timeframes used to define the timing of referral in the published literature makes it difficult to summarise and compare the results of various studies.

Moreover, although incorporation of risk prediction models such as the KFRE into day-to-day clinical practice comes with a lot of promise, their application may not be sufficient to capture individuals at high risk for progression who would benefit from timely referral to nephrology services. Consequently, more research is required to observe the impact of these prediction tools, such as the KFRE, on real life everyday practice. Crucially, variation across countries of the risk populations, availability of nephrology specialty care services, multidisciplinary care services, and practice patterns, would warrant a more tailored approach. This is further highlighted by the acknowledgement that inequities in care (and other social risks) impact referral practices and outcomes, which may not have been adequately described or explored in the papers reviewed.

## Conclusions

This review found that regardless of the time frame used to define early vs. late referral in relation to the start of KRT, evidence consistently revealed better outcomes for those referred early. However, the limitation of these findings lies in the fact that the definitions have all been done retrospectively, after the patients had already been referred to the nephrology services. The fact that it would be unlikely for ethical approval to be granted to randomise patients to late referral means that an RCT may never be conducted. Although the information gleaned from these studies can help to inform the length of time required for adequate predialysis care to mitigate adverse outcomes once dialysis starts, it may not shed light on when to refer these patients to the nephrology services.

Risk prediction equations are more suited for determining the timing of referral and hence can help with prospective planning. Thus, their development gives hope for the future, as it equips clinicians with the ability to accurately predict risk of clinically important outcomes. This will ensure referral of those individuals who stand the higher chance of benefiting from secondary care. Finding the risk threshold that would accurately predict outcomes such as the start of KRT or development of CVD would provide much needed guidance on the timing of referral from primary care to nephrology services.

### Future directions

Incorporation of risk prediction equations into referral guidelines is expected to better target the referral of individuals who are likely to benefit from specialist services, and hence ensuring more appropriate utilisation of scarce specialist workforce and capital resources. Efforts are also required to find a definition of early referral that is more universally acceptable to all stakeholders so that the referral process can be better streamlined and consistent to inform decisions on when to refer individuals with CKD to nephrology services in a timely manner.

An improvement in the promotion of primary care and specialist collaboration will facilitate the safe co-management in primary care of those individuals whose disease is unlikely to progress.

Further research is required to examine the safety and feasibility of referral of those individuals who are under the care of nephrologist services back to primary care once they have been stabilised and deemed suitable to be safely managed in primary care.

## Data Availability

No datasets were generated or analysed during the current study.
